# Supplementary material for: Mathematical basis for the assessment of antibiotic resistance and administrative counter-strategies
Source: PLoS One. 2020 Sep 3;15(9):e0238692. doi: 10.1371/journal.pone.0238692 (PMC7470328; doi:10.1371/journal.pone.0238692)
Supplement: S1 File — (PDF) [file pone.0238692.s002.pdf]

Supplementary information on data and R-code for statistical analysis for the manuscript entitled „Mathematical basis for the assessment of antibiotic resistance and administrative counter-strategies“ by Hans H. Diebner, Anna Kather, Ingo Roeder, and Katja de With

Data are available in RDS-Format and can be loaded using the attached R-Script (R version 3.6.1).

Content of data:

The data used in this analysis are from the University Hospital Carl Gustav Carus Dresden for the year 2017. Details included are: antibiotic substances measured as WHO-DDD, patient days, type of unit (medical, intensive care, OP), proportions of pathogens.

Data contains quarterly (from 2012 to 2017) recorded amounts (in DDD) of antibiotic consumption at the University Hospital Dresden, Germany, stratified by the functional units (medical, intensive care, surgical units) where the antibiotics have been consumed. Data also contains yearly recorded prevalences of resistent pathogens at the University Hospital Dresden, Germany. No patient data are involved, only aggregated consumption and prevalence data.

Origin of data:

Antibiotic consumption data was collected as part of a German national surveillance project (ADKA-if-DGI). Therefore, antibiotic orders from the medical departments, documented by the hospital pharmacy’s inventory management system and patient days recorded by the medical controlling were used (secondary data, i.e., no patient data involved).

Resistance data was collected from the hospitals microbiology department in an aggregated way:

We requested a database query (via Software Hybase) to get proportions of positive pathogen detections for several pathogen groups (e. g. Escherichia coli).

Data collected by the national surveillance project ADKA-if-DGI can be accessed at this website (http://www.antiinfektiva-surveillance.de).

Declaration:

Approvals of an IRB or ethics committee are not applicable for the data used here.
