# Supplementary material for: Mathematical basis for the assessment of antibiotic resistance and administrative counter-strategies
Source: PLoS One. 2020 Sep 3;15(9):e0238692. doi: 10.1371/journal.pone.0238692 (PMC7470328; doi:10.1371/journal.pone.0238692)
Supplement: Attachment — Submitted filename: Response to Reviewers.docx [file pone.0238692.s004.docx]

**Response to Reviewers**

**Manuscript PONE-D-19-25350R1**

Reviewer #2:

Both in their answer and in the updated manuscript, Diebner and collaborators clarified that they see the mathematical framework as the main contribution of their article, and the application to the hospital dataset as a mere example of what can be achieved using this framework.

While most of my objections regarding the analysis of this hospital dataset and the interpretation of the findings remain, I guess they should thus not be seen as a blocking point. Besides, I think the authors clarified many points regarding their methodology and the context of their work. Many of my methodological questions are now addressed, in the manuscript or in the answer. As a biologist, it is still hard for me to judge the interest of the measures proposed by Diebner and colleagues without seeing a convincing biological application. So all I can say is that the methods and the context are clearly described, especially with the clarifications made in this revision, and that the figures are easily understandable.

Still, I am surprised that the authors do not see the problem with correlating two time-series which are both highly autocorrelated (L400-408). The statistical analysis is not described in details so I may be wrong, but I looked at the code and it seems that the authors directly compute a Pearson correlation between the two vectors (each vector being a time series). If so, I think this is quite inappropriate (because the data points of each vector are not independently sampled from a given distribution). This is what I was trying to point in my initial review by stating that ``only time series are analysed''. I have nothing per se against the analysis of time series, but it should be done correctly with the appropriate statistical tools (for example analysing the residuals).

Answer:

The reviewer raised an important point regarding the correlation of time series. Yes, strictly speaking, Pearson’s correlation requires independent sampling. Usually, the correlation of two time series is analysed by applying a cross-correlation-function which uses a time-lagged version of one of the time series. Unfortunately, this is not applicable here due to the low sampling frequency and very short time series. Thus, our argument here is that Pearson’s correlation is sufficient to at least raise hypotheses. Arguably, this supports the idea of a proof-of-principle. Based on the proposed analysis using diversity measures it is planned to design a proper study and we think that also other researchers dealing with antibiotic stewardship will benefit from our mathematical framework. We added the subsection “Statistical analysis” at the end of the “Materials and methods” section to elaborate on these points.
